# Public awareness of autism spectrum disorder

**DOI:** 10.17712/nsj.2017.3.20160525

**Published:** 2017-07

**Authors:** Matar A. Alsehemi, Mahmoud M. Abousaadah, Razan A. Sairafi, Mohammed M. Jan

**Affiliations:** *From the Department of Pediatrics, Faculty of Medicine, King Abdulaziz University, Jeddah, Kingdom of Saudi Arabia*

## Abstract

**Objective:**

Examine the awareness of autism spectrum disorders (ASD) in our community which would help in early recognition and improved support of affected families.

**Methods:**

A focused 20-item questionnaire was designed to survey the public awareness and knowledge of ASD. Personal interviews were conducted during an ASD awareness day, which was organized in a major shopping mall on February 20, 2015 in Jeddah, Kingdom of Saudi Arabia.

**Results:**

A total of 259 individuals participated in the study with 47% being <30 years of age and 57% being females. Most participants (60%) were married, educated (68% university level), and employed (54%). When asked if they knew what autism is, 88% responded positively. However, when asked to rate their degree of knowledge, 41% felt that it is weak. Females and those older than 30 years of age were more likely to feel knowledgeable (p=0.04 for females and p=0.013 for those >30 years of age). Females were more likely to think that autistic children can be employed in the future (p=0.008), whereas males were more likely to think that autism is similar to mental retardation (p=0.005).

**Conclusions:**

The public awareness of ASD needs improvement. Areas for targeted education were identified to help improve the quality of life of autistic children and their families.

Autism spectrum disorder (ASD) is a relatively common neurodevelopmental disorder affecting up to 10/1,000 children worldwid.^1^ It is characterized by impaired social interaction, communication, and the presence of stereotyped activities.^2^ Early recognition is needed for early implementation of multidisciplinary treatment, which in turn improves the outcome.^3^,^4^ Enhanced public awareness is needed to prevent delays in the provision of such services.^5^ Improved awareness would also help in minimizing possible associated social stigma. These issues have received limited study in our region with a critical gap in ASD research in developing countries.^6^,^7^ Such information is needed for planning adequate autism education and awareness campaigns. We hypothesized that there are considerable misconceptions and lack of knowledge about ASD in our community, which we aim to examine in this study.

## Methods

A focused 20-item questionnaire was designed to survey the public awareness and knowledge of ASD. Demographic variables and key questions on ASD awareness and knowledge were selected based on the available published literature and was initially drafted in English and subsequently translated into Arabic (**Table 1**). Personal interviews were conducted randomly during an ASD awareness day, which was organized in a major shopping mall (Red Sea mall) on February 20, 2015 in Jeddah, Kingdom of Saudi Arabia. The nature of the study and questionnaire were explained to the participants before voluntarily participating in the study. Only adult participants were included. An assigned coauthor conducted the interviews and individually assisted them to complete the questionnaires. These were conducted before providing the information from the educational materials, which was available during the campaign. The identity of the participants was not requested to ensure privacy and encourage accurate response. The study design and questionnaire were approved by King Abdulaziz University hospital ethics. Data were collected in Excel sheets and statistical analysis was performed using Statistical Package for the Social Science 22 (IBM Corp., Armonk, NY, USA). Descriptive analyses were performed and the variables were examined using chi-square test. Statistical significance will be defined as *p*-values < 0.05.

**Table 1:** Key questions about public ASD awareness and attitudes. (N=259)

Questions	Positive Answer n (%)
Do children with autism have limitations in their social skills?	237 (92)
Do children with autism have language and communication problems?	205 (79)
Are you aware about any special centers for children with autism?	190 (73)
Can the child with autism be employed in the future?	183 (71)
Children with autism are at increased risk for epilepsy?	155 (60)
Are there any medical treatments for children with autism?	126 (49)
Can the child with autism attend regular school?	104 (40)
Autism is related to using electronics?	91 (35)
Is autism the result of improper parental upbringing of the affected child?	64 (25)
Is the society aware on how to interact with autistic children?	61 (24)
Autism is similar to mental retardation?	49 (19)
Is autism an organic disease, similar to cerebral palsy?	43 (17)

## Results

A total of 259 participants participated in the study. The sample had a variable age distribution with 47% being <30 years of age. Both males and females participated with 57% being females. Most participants (60%) were married, educated (68% university level), and employed (54%). Only 19% of the participants were still studying. When asked if they knew what autism is, 88% responded positively. However, when asked to rate their degree of knowledge on the disorder, only 9% felt that it is strong and the majority (41%) felt that it is weak. The remaining 50% rated their knowledge as “medium”. Females and those older than 30 years of age were more likely to feel knowledgeable about autism (*p*=0.04 for females and 0.013 for those >30 years of age). Answers to specific knowledge and awareness questions are summarized in **Table 1**. Specific misinformation or misconceptions did not further correlate with age, social status, education, or occupation. However, females were more likely to think that autistic children can be employed in the future when compared to males (*p*=0.008), while males were more likely to think that autism is similar to mental retardation (*p*=0.005).

## Discussion

Our study suggests that the public awareness about ASD needs improvement. Considerable misconceptions and misinformation were identified, which can be targeted by focused educational campaigns.^8^ Such initiatives should facilitate the development of systematic and sustainable solutions for enhancing autism awareness and hence develop effective and sustainable public health solutions for disseminating beneficial information to affected families.^9^ There is some evidence that such limited awareness and understanding about autism involves not only the public but also teachers and health care professionals.^10^

Although most of our participants felt that they had prior knowledge about ASD, such knowledge was weak in most of them. This was further confirmed by our focused questions, which uncovered specific misconceptions (**Table 1**). These included lack of knowledge of any medical treatments, thinking that these children should not attend regular schools, and believing that such a disorder can be related to electronics or parental upbringing practices. On the other hand, many participants correctly recognized important features of the disorder and were able to recognize the need for enrollment in specialized autism centers (**Table 1**). Interestingly, females were more knowledgeable about the disorder when compared to males, which may reflect improved exposure or their interest. As expected, older participants (>30 years of age) were more likely to feel knowledgeable about autism reflecting their improved experience when compared to younger participants. However, specific misinformation or misconceptions did not further correlate with social status, education, or occupation.

There are some limitations to our study. First, our study sample was relatively small, however, we enrolled participants with various ages and equal gender distribution. Secondly, our sample was collected from one region so it may not be representative of the Saudi public. It reflects the working class with higher educational levels, likely the result of the included shopping mall, which is located in an upscale part of the city. This may limit the ability to generalize from our findings. In addition, our questionnaire was brief and not detailed. We avoided making it longer to avoid any practical difficulties in completing it on time in such a public setting. Limited such literature is available in our region to investigate the burden of the disease on the society.^11^ More detailed research is needed to better identify the burden and social limitations created by autism in our region.

In conclusion, the Saudi public awareness of ASD needs improvement. Some people still believe that it is related to electronics or parental upbringing practices. While others think that it is similar to mental retardation or cerebral palsy. We were able, therefore, to identify targeted areas for focused education, particularly using the media, in order to minimize associated social stigma.^12^ Such educational campaigns should target the general public and focus on means to help enhance the quality of life of children with autism and their families. A more informed community will certainly be more tolerant to these unfortunate children.
